# The role of social capital in the impact of multiple shocks on households’ coping strategies in underdeveloped rural areas

**DOI:** 10.1038/s41598-024-65206-x

**Published:** 2024-06-20

**Authors:** Yuying Yang, Yanfang Huang, Jiaqi Huang, Fengying Nie

**Affiliations:** 1 School of Agricultural Economics and Rural Development, Renmin University of China, Beijing, 100872 China; 2 Agricultural Information Institute, Chinese Academy of Agricultural Sciences, Beijing, 100081 China; 3 Institute of Plant Protection, Chinese Academy of Agricultural Sciences, Beijing, 100193, China

**Keywords:** Multiple shocks, Social capital, Households’ coping strategies, Underdeveloped rural areas, Natural disasters, Environmental social sciences, Natural hazards

## Abstract

Social capital has long been recognized as a facilitator of socio-economic development. However, the role of social capital in enhancing resilience to multiple shocks in rural China remains insufficiently explored. This study focus on the resilience of households that have recently get rid of poverty and reside in underdeveloped rural areas of China. Unlike previous studies, the article incorporates multiple shocks, social capital, and households’ coping strategies into a research framework at the micro level. This study systematically analyses the multiple shocks experienced by households, their coping strategies, and further explores the mediating role of social capital. Utilizing two waves of a rural household panel survey data collected in six underdeveloped counties in 2015 and 2018 in China, we present four key findings. Currently, households primarily contend with drought, illness of family members, and the high costs of agricultural inputs as the main shocks. Their predominant coping strategy is reducing consumption. Importantly, social capital exhibits a mediating effect, accounting for 9.8% of the impact of multiple shocks on households’ coping strategies. Notably, natural disasters significantly diminish the informal functions of social capital. While social capital exerts a full mediating effect in non-agricultural households, this effect is not observed among others. This study contributes to a better understanding of the dynamics and specificities of social capital in vulnerable rural areas. Additionally, the findings provide policymakers with practical insights regarding differentiated and preemptive risk governance approaches.

## Introduction

Rural livelihoods in developing countries are disproportionately vulnerable to multiple shocks and stresses^1^. Shocks are events with significant negative welfare effects that can be environmental (i.e., climate variability, low fuelwood, or limited fruit tree availability), social (i.e., death or injury of a family member), or economic (job loss, reduction in government grants, or decreased remittances). Negative shocks can exacerbate human vulnerability, which can subsequently adversely affect rural livelihoods. Globally, extreme weather and natural disasters are deemed to pose the biggest risks, and the annual economic loss caused by disasters accounts for 0.1–0.5% of global gross domestic product^2^. For example, extreme weather conditions have been shown to reduce agricultural producers’ mean yields and increase output variance in developing countries^3^.

Despite China’s noteworthy achievements in poverty alleviation in recent decades, a risk of poor households returning to absolute poverty remains. China has a complex disaster-prone environment, prominent three-step terrain, and the significant “geographic coupling” characteristics^4^, which has led to severe losses from natural disasters. Social shocks such as major diseases and labor shortages can seriously diminish households’ income^5^. In addition, economic shocks such as high agricultural input costs and rising food and fuel prices threaten to farm households’ livelihoods due to the nature of agricultural production, elasticity of demand for agricultural products, and changes in the external environment^6^. A recent data indicates that in underdeveloped areas of China, 19.45% of farm households still face severe risk of a large-scale return to absolute poverty^7^, indicating that uncertainties remain after poverty alleviation in underdeveloped areas, making farm households vulnerable to sliding below the poverty line. Therefore, Chinese No. 1 Central Document for 2023 cited one of the current priorities as preventing vulnerable households’ return to poverty.

Rural society has developed a mutual support system founded on social capital for guarding and buffering against shocks^8^. Social capital is the resources embedded in social structures that can be acquired or mobilized based on purposeful actions, including trust, reciprocity, political connections, and other types of social networks^9^. However, rural decline has become a global issue along with urbanization and industrial development^10^. In China, there will be 170 million migrant workers out of the country in 2022^11^. As people leave rural areas, labor shortages and possibly resulting recession, social degradation, and shrinking local markets challenge the mutual system of social capital. The bonding and bridging aspects of social capital are vulnerable to rural depopulation^12^. In the process, villages tend towards individualism, lose social cohesion, and become socially and economically isolated, resulting in their inability to cope with shocks^13^. In contrast, other villages have been able to mobilize internal and external resources to bring about locally initiated change that benefits the wider community and adapts to changing circumstances. For example, the revival of the mountain village of Åre in north Sweden and Xiaoguan village in China’s Hebei Province have demonstrated the important role of local social capital in enhancing the endogenous development capability of the community^14^. Therefore, The role of social capital is not entirely negative in remote rural areas, as shown in the positive examples deviating from the general negative pattern.

It should be emphasized that the role of social capital in rural development remains a relatively under-explored area of research, especially for those less developed rural areas in transition and revitalization. Improving low-income households’ livelihood resilience against challenges and shocks has been emphasized to reduce the risks of reverting to poverty in China. Geographically remote from urban centers and with limited formal safety nets provided by the state or the market, these areas rely on mutual aid systems of social capital to withstand multiple shocks but also face the challenge of rural depopulation. Moreover, increasingly frequent and intense shocks, may overburden households’ existing coping strategies and push households and communities beyond their capacity to cope^15,16^. Therefore, the shock-resistant role of social capital needs to be revisited to increase rural resilience^17^. In light of this context, our study endeavors to address the following research questions: What are the main shocks faced by households? What coping strategies do they employ? Can social capital play a role in mitigating multiple shocks? Who is unable to utilize social capital in response to shocks?

This study contributes to the related literature in four ways. Firstly, we integrate multiple shocks, social capital, and coping strategies into a framework that considers the role of social capital in the impact of multiple shocks on the coping strategies of households in underdeveloped areas. From a policy perspective, there is a growing recognition that a sustainable resilience system necessitates the establishment of diverse arrangements, combining formal and informal safety nets. These arrangements offer alternative resources for individuals with low incomes to access when they face critical needs^18^. Despite this recognition, there is a lack of clear policies and concrete information regarding the role of social capital in enhancing the resilience of impoverished households amidst accelerated urbanization. This research addresses this gap by scrutinizing the role of local forms of social capital in fostering resilience. Our research reveals that social capital has a mediating effect on the impact of multiple shocks on households’ coping strategies, which can explain the mechanism by 9.8%.

Secondly, it is one of the few studies to analyze the effects of multiple shocks on household coping strategies. While most previous studies separately investigated the impact of a single type of shock on households, our study quantifies the effects of different types of shocks on household coping strategies. Shocks encompass complex forms of natural, economic, and social effects^19^, and understanding the compounded impacts of different types of shocks is crucial. This approach allows us to identify less insurable shocks, such as drought, illness of family members, and the high costs of agricultural inputs, offering valuable insights for policymakers and development practitioners to prioritize and design appropriate social programs.

Thirdly, we classify the households in underdeveloped areas according to different types of livelihoods. Recognizing that social capital is not uniformly distributed among community members, and social ties can be a stratified and differentiated resource^20,21^, we explore the varying mediating effects of social capital among different groups. Our study shows that social capital exerts a full mediating effect in non-agricultural households, this effect is not observed among others. This investigation delves into the controversy surrounding whether social capital serves as the capital of low-income individuals.

Finally, we use two waves of survey data (2015 and 2018) from 114 villages among 2740 farm households in six underdeveloped counties in China. Therefore, further contributions of this study in terms of data are that the sample size is representative of households living in underdeveloped rural areas, and the two survey waves allow temporal comparability. The research provides the latest strong evidence for understanding the effectiveness and limits of social capital to shocks.

The remainder of this paper is organized as follows. Section “Literature and hypothesis” presents literature review of the theoretical and empirical basis of the subject matter. Section ”Data and methods” introduces the data sources, variables, and empirical methods used in this study. Section ”Result” details the results of our analysis, Section ”Discussion” offers a discussion of the findings, and Section ”Conclusions and policy recommendations” concludes.

## Literature and hypothesis

**Figure 1:**
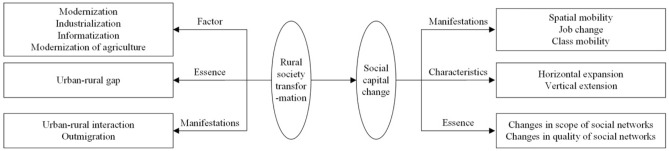
Theoretical framework of social capital.

Farm households around the world are increasingly exposed to external and internal shocks. Enhancing the resilience of farm households to frequent disturbances is critical to promoting the sustainability of their livelihoods and the revitalization of rural areas. Strong social capital enables local populations to acquire the capacity to learn from and adapt to changing environments, which can lessen the impact of challenges and stresses and hasten the recovery process. However, the influence of social capital is not entirely positive. While some households can leverage social capital for risk-sharing, others may be less capable of withstanding risks. This dynamic is closely linked to changes in social capital prompted by the ongoing transformation of rural society (Fig. 1).

Influenced by industrialization, informatization, urbanization, and the modernization of agriculture, rural areas are transitioning from traditional to modern societies^12^. This shift, originating from the urban–rural divide, is marked by outmigration from rural areas. As rural social transforms, social capital undergoes a cycle of inheritance and erosion. With the urbanization of farmers, their children attending college, capital influx into rural areas, and government officials engaging at the grassroots level, household social networks are expanding not only horizontally but also vertically^22^. For example, technologies have integrated economic activities into dense networks^23^. As increasing number of economic-driven factors are being incorporated into people’s norms and values, which, in turn, affect their decisions. Some farmers have adapted to new production methods and lifestyles, including industrial upgrades, thereby widening their “circle of friends” and greatly enhanced their resilience to multiple shocks. Conversely, some farmers struggle to mobilize resources, become socially and economically isolated, and face challenges in establishing mutual assistance mechanisms, resulting in limited potential for direct risk-sharing.

We seek to deepen our understanding of the role of social capital in household resilience against shocks, drawing on theories and lessons learned about multiple shocks, coping strategies, and social capital.

### The impact of multiple shocks on households’ coping strategies

The risk-sharing theory claims that idiosyncratic shocks should be absorbed by the cooperation between households within the same risk-sharing network. A number of studies examine directly the impact of a specific type of shocks on household consumption (e.g., health shocks, weather-related shocks)^24–26^. However, most of these studies do not consider coping strategies. A limited number of studies analyze various types of shocks, but the results vary across countries.

In case of shocks, households in poor rural areas rely on different levels of risk coping mechanisms to overcome potential losses. Specifically, since households often lack effective market-based measures for managing shocks, they tend to arrange self-insurance by altering production and consumption practices. Households’ measures are hierarchical, generally preferring reduced consumption and savings to address losses^27^; however, if savings are insufficient, households can often borrow from friends and relatives. When borrowing is a challenge, households “self-exploit” their labor force by working longer hours outside the home and other income-generating activities^28^. If financial difficulties are still not effectively alleviated, households will then sell assets in exchange for income^29^. Other coping strategies include migration^30^, and agricultural adaptation^31^ and on-farm diversification^32^. In summary, households employ rational shock mitigation strategies involving multiple choices, with costs and benefits reflecting the weighted averages of these options.

To cope with shocks (both covariate and idiosyncratic shocks), households may adopt self-insurance coping strategies or risk-sharing strategies^33^. Risk-sharing strategies refer to the informal arrangement between households in a community or a group to support each other to cope with shocks (e.g., assistance, gift, credit). Self-insurance strategies refer to the reallocation of households’ resources (e.g., depleting savings, selling assets, using child labor)^34,35^. Depending on the nature of shocks, households may have different choices and combinations of coping strategies. Households tend to use risk-sharing strategies to protect their consumption against idiosyncratic shocks^36^. Natural disasters are covariate shocks that tend to affect the whole community, and group risk-sharing strategies based on social networks may be affected. In addition, households tend to use multiple shock-coping strategies rather than relying solely on a single strategy. As shocks are not the weighted average of these choices, employing several strategies can mitigate the impact of multiple shocks, leading to reduced household loss^37^. Based on the above analysis, we propose the first hypothesis.

#### H1

Multiple shocks can increase households’ coping strategies in underdeveloped rural areas.

### The impact of social capital on households’ coping strategies

Social capital is a low-cost form that complements households’ coping strategies. In Chinese rural society, characterized by blood ties, kinship, and local connections, community members closely communicate and trust each other, leading to low transaction costs and smooth information transmission^38^. The following theoretical model of households’ coping mechanisms is constructed based on existing research^39^.

We begin by assuming that: (1) One or more risk-sharing groups exist within the village. (2) Intra-rural members (in terms of households) face multiple shocks, but no single shock can affect the marginal effect function in each period. (3) Information can be adequately communicated between rural households. (4) Consumption and leisure fulfillment are separable and they are independent of each other. (5) Preferences satisfy additivity in the individual dimension as well as in the time dimension, and households have the same time discount rate. (6) Each household in the village has the same utility function in the form of Eq. (1).

The utility function of the representative household is:

1 $${U}_{it}=\sum_{t=1}^{T}{\beta }^{t}\sum_{s=1}^{S}{\pi }_{s}{u}_{i}\left({c}_{ist}\right)$$

Specifically, $i=1, 2, 3, \dots , N$, denote the first household in the village $i$ household, the village is assumed to consist of $N$ households. $t (t=1, 2, 3,\dots , T)$, represents the different period subscripts of the household families, and $T$ is the total duration. $S$ represents uncertainty, indicating that the household $i$ may occur $S$ situations (status of nature) during the period. $s (s=1, 2, 3,\dots , S)$, representing a specific occurrence of a status with a probability of occurrence of ${\pi }_{s}$, is public information that is known to all households. ${U}_{it}$ is the utility function of the household, and ${c}_{ist}$ is the utility function of the household $i$ has at each moment $t$ and status $s$, where the household’s utility function $U (\cdot )$ is quadratically differentiable and satisfies $\frac{\partial u}{\partial c}>0$, $\frac{{\partial }^{2}u}{\partial {c}^{2}}>0$ , and $\underset{c\to 0}{\text{lim}}\frac{\partial u}{\partial c}=+\infty$. $\beta$ is the same time discount rate for all farmers.

As mentioned above, the Pareto allocation of risk within the village can be obtained by maximizing the weighted sum of the expected utilities of all farm households, and Pareto-efficient risk sharing among members within the village can be achieved by maximizing the weighted utility function of Eq. (2), where ${\omega }_{i}$ is the weight of the household $i$ and satisfies $0<{\omega }_{i}<1$. $\sum_{i=1}^{N}{\omega }_{i}=1$.

2 $$MAX\left\{\sum_{i=1}^{N}{\omega }_{i}\sum_{t=1}^{T}{\beta }^{t}\sum_{s=1}^{S}{\pi }_{s}{u}_{i}\left({c}_{ist}\right)\right\}$$

3a $$s. t. \sum_{i=1}^{N}{c}_{ist}=\sum_{i=1}^{N}{y}_{ist}, \forall s, t$$

3b $${c}_{ist}\ge 0, \forall i, s, t$$

Equation (3a) is for each period $t$ each possible status that can occur $s$ under the resource constraints within the village network, and Eq. (3b) is the non-negative constraint. ${y}_{ist}$ is the household income constraint the household $i$ has in the period $t$ and status $s$. Construct the Lagrange function according to Eqs. (2) and (3a), with ${\lambda }_{i}$ is the Lagrange multiplier.

4 $$L=\sum_{i=1}^{N}{\omega }_{i}\sum_{t=1}^{T}{\beta }^{t}\sum_{s=1}^{S}{\pi }_{s}{u}_{i}\left({c}_{ist}\right)+\sum_{i=1}^{N}{\lambda }_{i}\left({y}_{ist}-{c}_{ist}\right)$$

Solving the partial derivatives for household $i$ and household $j$ within the village, respectively, yields Eqs. (5a) and (5b).

5a $$\frac{\partial L}{\partial {c}_{ist}}={\omega }_{i}{\beta }^{t}{\pi }_{s}{u}_{i}{\prime}\left({c}_{ist}\right)-{\lambda }_{i}{\beta }^{t}{\pi }_{s}=0$$

5b $$\frac{\partial L}{\partial {c}_{jst}}={\omega }_{j}{\beta }^{t}{\pi }_{s}{u}_{j}{\prime}\left({c}_{jst}\right)-{\lambda }_{j}{\beta }^{t}{\pi }_{s}=0$$

According to Eqs. (5a) and (5b), and ${\lambda }_{i}={\lambda }_{j}$, we can obtain:

6 $$\frac{{u}_{i}{\prime}\left({c}_{ist}\right)}{{u}_{j}{\prime}\left({c}_{jst}\right)}=\frac{{\omega }_{j}}{{\omega }_{i}}, \forall i, j, s, t$$

For any household within the village, Eq. (6) in any period $t$ and in any status $s$ holds. The marginal utility of all members within the village network varies in the same direction, as does their level of consumption. Therefore, in any status $s$ of the village, the marginal utility of any household $i$ is a monotonically increasing function of the average marginal utility of all members of the village household. This implies that any farmer’s consumption is a monotonically increasing function of the average consumption of all households in the village network. To visualize this result more concretely, it is assumed that each household in the village has the same constant absolute risk aversion utility function (CARA) of the following form:

7 $${u}_{i}\left({c}_{ist}\right)=-\frac{1}{\sigma }{e}^{-\sigma {c}_{ist}}$$

Applying this utility function to the first-order condition for optimization, Eq. (6), yields:

8 $$\frac{{u}_{i}{\prime}\left({c}_{ist}\right)}{{u}_{j}{\prime}\left({c}_{jst}\right)}=\frac{{e}^{-\sigma {c}_{ist}}}{{e}^{-\sigma {c}_{jst}}}=\frac{{\omega }_{j}}{{\omega }_{i}}$$

Taking the logarithm of Eq. (8) and performing the transformation yields equation:

9 $${c}_{ist}={c}_{jst}+\frac{1}{\sigma }\left(\mathit{ln}{\omega }_{i}-\mathit{ln}{\omega }_{j}\right)$$

At any given time $t$, Eq. (9) holds for all households and hence there is:

$${c}_{ist}={c}_{1st}+\frac{1}{\sigma }\left(\mathit{ln}{\omega }_{i}-\mathit{ln}{\omega }_{1}\right)$$

10 $${c}_{ist}={c}_{2st}+\frac{1}{\sigma }\left(\mathit{ln}{\omega }_{i}-\mathit{ln}{\omega }_{2}\right)$$

$${c}_{ist}={c}_{Nst}+\frac{1}{\sigma }\left(\mathit{ln}{\omega }_{i}-\mathit{ln}{\omega }_{N}\right)$$

Averaging Eq. (10):

11 $${c}_{ist}=\frac{1}{N}\sum_{j=1}^{N}{c}_{jst}+\frac{1}{\sigma }\left(\mathit{ln}{\omega }_{i}-\frac{1}{N}\sum_{j=1}^{N}\mathit{ln}{\omega }_{j}\right)$$

Further differencing of Eq. (11), we yield Eq. (12):

12 $$dc_{ist} = d\overline{{C_{st} }} , {\text{ among others: }}\overline{C} = \frac{1}{N}\mathop \sum \limits_{j = 1}^{N} c_{jst}$$

Where Eq. (11) clearly shows that to household $i$, whose consumption depends only on the average consumption of all households in the village and increases with the average consumption of the village. Equations (11) and (12) also show that if a Pareto allocation of risk can be achieved within the village, where any temporary shock can be fully integrated and shared within the village.

This implies that the total income of the village will be distributed within the village according to the actual needs of individual households. If a household suffers a shock such that the marginal utility of consumption is higher than that of other households, the intra-village risk-integration mechanism will utilize reciprocal income transfers in the cross-section to eliminate this difference, so that the shocks faced by households can be fully integrated within the village. Based on the above analysis, we propose the second hypothesis.

#### H2

Social capital can increase the coping strategies of farm households.

### The mediating effect of social capital

Social capital operates in three primary ways. Firstly, social capital facilitates labor mobility. Many empirical studies from developing countries and regions have found that social capital, especially social networks, facilitates the mobility of rural surplus labor^40^. The mechanisms through which social capital plays a role are mainly in facilitating the transmission of information and the provision of social insurance, as well as raising wages to a certain extent^41^. Notably, “Guanxi” networks significantly influence access to non-farm jobs and play a vital role in information flow within the labor market^42^. Social capital is a key source that can help residents share physical, financial, and social resources in drought conditions. Even in normal conditions, strong social networks can help the members of a community to find jobs^43^.

Secondly, social capital can provide financial support. Under the existing conditions of incomplete property rights in rural China, social networks are important for farmers in balancing cash flow and alleviating liquidity constraints. While the scale and role of farmers’ private lending behaviors may have weakened with social and economic transformations, social network-based private lending remains significant in fulfilling rural finance needs^44^. Moreover, local society’s ability to provide essential commodities through social relations becomes evident in critical conditions^45,46^, demonstrating how social capital helps individuals navigate life changes and challenges.

Thirdly, social capital can facilitate the adoption of new technologies. The diffusion of new technologies in poor rural areas is closely related to local social networks and trust. When natural disasters occur, farmers transmit information through social networks to reduce transaction costs and adopt timely agricultural technologies that facilitate risk diversification^47^. A study in rural Mozambique also found that social networks can be used as a means of information transfer. Decisions on whether or not to adopt new technologies depend on the decisions of other farmers in the social network^48^.

Therefore, this study posits that social capital increases households’ coping strategies through stabilizing income and sustaining production and operations following multiple shocks. Based on the above analysis, we propose the third hypothesis.

#### H3

Social capital has a mediating effect on the impact of multiple shocks on households’ coping strategies.

**Figure 2:**
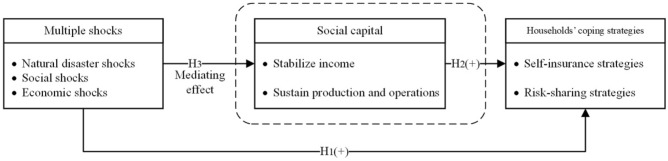
Theoretical analysis of multiple shocks, households’ coping strategies and social capital.

Figure 2 is the diagram of multiple shocks, households’ coping strategies and social capital.

## Data and methods

### Data

This study conducted a face-to-face household survey involving 2740 households in 119 villages across six underdeveloped counties in Southwest China. The survey was carried out by the Agricultural Information Institute of the Chinese Academy of Agricultural Sciences (CAAS) in 2015 and 2018. The dataset, rich in detailed indicators covering multiple shocks (22 types), coping strategies (19 types), and social capital, serves as a robust foundation for achieving the study’s research objectives.

**Figure 3:**
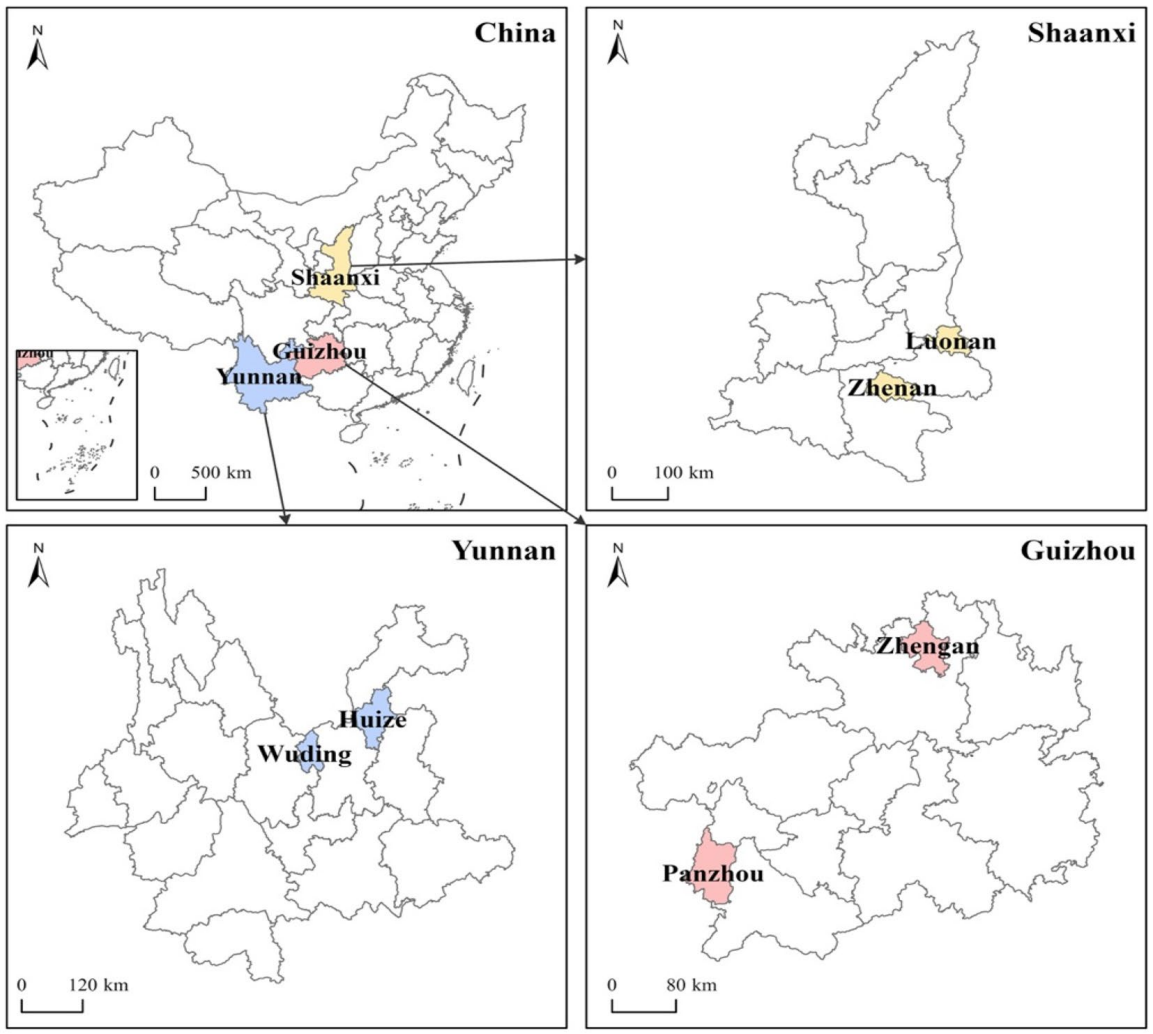
Location map of sample provinces and counties (This figure is generated by ArcGIS pro 3.0.2 software. URL link: https://www.esri.com/en-us/arcgis/products/arcgis-pro/overview).

The samples selection process involved four steps. First, based on the National Plan for Poverty Reduction from 2011 to 2020, the Chinese government identified 592 poor counties, most of which are characterized by challenging natural conditions and disaster-prone areas. Second, six counties were selected based on the willingness of residents to cooperate and the high prevalence of small-scale farming: Luonan and Zhen’an County in Shaanxi Province, Wuding and Huize County in Yunnan Province, and Pan and Zheng’an County in Guizhou Province (Fig. 3). Third, in each of the six counties, 19 villages were selected using the probability proportional to size (PPS) method. Finally, 12 households within each sampled village were randomly chosen, and residents were interviewed face-to-face, resulting in a total sample of 1368 households for empirical analysis.

The survey covered self-reported household information on demographics, livelihood capital, multiple shocks, and coping strategies. Trained enumerators posed the questions and recorded the respondents’ answers using electronic questionnaire equipment.

The sample size is 2740, with 1368 observations in 2015 and 1372 in 2018, resulting in a 66.7% comparable sample revisit rate. The proportion of poor households in the sample was 35.2%, and multiple shocks occurred in 67.5%.

To improve our empirical estimations, we cleaned the data rigorously in three steps. First, we deleted samples that reported missing values for variables representing multiple shocks and coping strategies. Second, samples with missing or abnormal values on social capital were excluded. Third, we excluded samples that reported missing or abnormal answers on the control variables. This resulted in a final sample size of 2740, with a 66.7% comparable sample revisit rate. The proportion of poor households in the sample was 35.2%. Multiple shocks occurred in 67.5%.

### Variable description

The independent variable is multiple shocks (Table 1). As noted earlier, shocks are events with significant negative welfare effects and can be environmental, social, or economic in nature^49,50^. Following Morduch^51^ and Nie^52^, multiple shocks are categorized into natural disaster shocks and unnatural disaster shocks. Unnatural disaster shocks are further divided into social and economic shocks. Households were queried about the occurrence of drought, flood, hail, frost, and other disasters in the past 12 months, assigning a value of 1 if the response was affirmative. The variable multiple shocks is an aggregate value.

**Table 1:** Multiple shocks for sample households according to category statistics.

Shock category	Full sample (n = 2740)		2015 (n = 1368)		2018 (n = 1372)	
	Freq	%	Freq	Freq	%	Freq
1. Natural disaster shocks						
Drought	710	25.91	507	37.06	203	14.80
Heavy rainfall/flood	486	17.74	270	19.74	216	15.74
Hail	184	6.72	106	7.75	78	5.69
Frost/snowstorm	253	9.23	135	9.87	118	8.60
Rare hot weather	132	4.82	86	6.29	46	3.35
Wind damage	393	14.34	239	17.47	154	11.22
Mudslide	75	2.74	53	3.87	22	1.60
Fire	13	0.47	9	0.66	4	0.29
Severe crop diseases	170	6.20	122	8.92	48	3.50
Severe livestock diseases	92	3.36	67	4.90	25	1.82
Earthquake	65	2.37	63	4.61	2	0.15
Wildlife destruction	124	4.53	89	6.51	35	2.55
2. Unnatural disaster shocks						
2.1 Social shocks						
Lack of workforce	371	13.54	270	19.74	101	7.36
Household member illness	580	21.17	375	27.41	205	14.94
Death of a capable member	16	0.58	9	0.66	7	0.51
Death of other members	31	1.13	13	0.95	18	1.31
Conflict	4	0.15	3	0.22	1	0.07
2.2 Economic shocks						
High cost of agricultural inputs	243	8.87	201	14.69	42	3.06
Decrease in member income	152	5.55	123	8.99	29	2.11
Theft of property	7	0.26	2	0.15	5	0.36
Theft of raised livestock	9	0.33	5	0.37	4	0.29
Rising food and fuel prices	178	6.50	145	10.60	33	2.41

Overall, the prevalent shocks faced by households include drought, illness of family members, and the high cost of agricultural inputs. Notably, the incidence of natural, social, and economic shocks declined from 2015 to 2018, but over half of the respondents still experienced shocks in 2018. Natural disasters exhibited the highest incidence with a slower decreasing trend. Social shocks were primarily linked to a lack of workforce, while the high cost of agricultural inputs was the most frequent economic shocks.

The dependent variable is households’ coping strategies (Table 2), derived from the question: “When faced with a specific shock, what are the main coping strategies your family took?” The aggregated number of countermeasures indicates the flexibility of shock coping and resilience.

**Table 2:** Coping strategies of sample households (%).

Coping strategies category	Full sample (n = 2740)	2015 (n = 1368)	2018 (n = 1372)
Purchase of the same type of low-priced food	10.15	10.53	9.77
Reduce food consumption with rising prices	9.93	10.60	9.26
Reduce meat consumption	6.20	6.51	5.90
Reduce eggs consumption	1.50	2.34	0.66
Reduce milk consumption	0.33	0.37	0.29
Reduce fruits consumption	1.93	1.54	2.33
Reduce vegetable consumption	1.72	2.27	1.17
Reduced spending on clothing	7.01	8.55	5.47
Reduced spending on medical care	0.84	0.66	1.02
Reduced spending on education	0.15	0.07	0.22
Reduced spending on visiting friends and relatives	3.54	3.58	3.50
Reduce spending on agricultural inputs	3.69	5.70	1.68
Use savings	13.36	15.42	11.30
Borrow money	15.00	19.66	10.35
Work outside the home	4.09	8.19	0.00
Sell property	0.58	0.80	0.36
Sell or eat livestock in advance	1.93	2.92	0.95
Sell farm equipment, seeds, or other inputs	0.11	0.15	0.07
Sell agricultural products in advance	1.06	1.32	0.80

Data reveals that 47% of farm households chose to reduce consumption in various categories (food, clothing, medical care, and education) when facing multiple shocks. Such reduction may limit future investment, impacting human capital. Borrowing money was the primary practice for 15% of households, while 4.9% chose working outside the home. This suggests generally single and inflexible coping strategies, with the top two measures focusing on consumption reduction and borrowing money. Research indicates that a diverse set of strategies reduces risk, implying that inflexible coping strategies correlate with higher risk exposure.

The mediating variable, social capital (Table 3), is measured through household participation in community organization and access to social network support. Synthesizing prior studies^53^ and aligning with the context of undeveloped rural areas in China, participation criteria include household members being village leaders and joining specialized farmers’ cooperatives. Access to social network support encompasses gift expenses, informal borrowing, gift income, and information support, measured by internet expenses. Each index, treated as a dummy variable (Yes = 1, No = 0), contributes equally to construct a social capital score—a proxy variable where a high score signifies abundant social capital.

**Table 3:** Dimensions, assignment rules, and weights of social capital.

Social capital dimension	Assignment rules	Weights
Internet expenses	Yes = 1, No = 0	1/6
Family members as village leaders	Yes = 1, No = 0	1/6
Joined specialized farmers’ cooperatives (planting, breeding, and other cooperatives and associations)	Yes = 1, No = 0	1/6
Gift expenses	Yes = 1, No = 0	1/6
Informal borrowing and loans	Yes = 1, No = 0	1/6
Gift income	Yes = 1, No = 0	1/6

To diminish the influence of confounding factors, this study controls for variables of age, education level, number of household labor force, household arable land, household assets ownership, formal insurance, household debt, and bank loans, referencing previous research^52^ (Table 4).

**Table 4:** Variable definitions and descriptive statistics (n = 2740).

Variables	Description and Measure	Mean	SD
Coping strategy	Number of coping strategies for shocks	0.95	1.77
Multiple shocks	Households suffered from shocks (times)	1.57	1.77
Social capital	Scores for the six dimensions of equally weighted assignments presented in Table 4	0.51	0.34
Age	Householder’s age	52.93	11.20
Education level	Householder’s years of education	6.41	3.55
Number of household labor force	Number of people in the household aged 18–65	3.17	1.30
Household arable land status	Actual arable land area per capita (mu)	1.15	2.57
Household assets ownership	The number of assets owned by the household as a proportion of the options listed	0.23	0.09
Formal insurance	Whether a household has social insurance or other insurance (no = 0, yes = 1)	0.70	0.46
Household debt	Whether the household has debt (no = 0, yes = 1)	0.59	0.49
Bank loan	Whether a loan is from a bank (no = 0, yes = 1)	0.07	0.26

### Empirical model

The following panel data model is constructed to analyze the effects of multiple shocks and social capital on households’ coping strategies and mechanisms.

13 $${cope}_{i,t}={a}_{0}+{a}_{1}{shock}_{i,t}+{a}_{2}c{ontrol}_{1}+{\varepsilon }_{i,t}$$

14 $${socialscore}_{i,t}={b}_{0}+{b}_{1}{shock}_{i,t}+{b}_{2}c{ontrol}_{2}+{u}_{i,t}$$

15 $${cope}_{i,t}={c}_{0}+{c}_{1}{shock}_{i,t}+{c}_{2}{socialscore}_{i,t}+{c}_{3}c{ontrol}_{3}+{v}_{i,t}$$

In Eqs. (13)–(15), ${cope}_{i,t}$ is the number of coping strategies adopted by farm household $i$ in year $t$, ${shock}_{i,t}$ is the number of multiple shocks suffered by farm household $i$ in year $t$, and ${socialscore}_{i,t}$ is the social capital score of farm household $i$ in year $t$. The specific meaning and calculation method are presented in Table 4. $c{ontrol}_{1}$–$c{ontrol}_{3}$ are the relevant control variables, with other variables hypothesized to affect the dependent variables. ${\varepsilon }_{i,t}$, ${\mu }_{i,t}$, and ${v}_{i,t}$ are stochastic disturbance terms.

This study references Baron and Kenny^54^ and Wen^55^ to examine the mediating effect of social capital on the impact of multiple shocks on households’ coping strategies. Specifically, in Eqs. (13)–(15), if the coefficients to be estimated (${a}_{1}$, ${b}_{1}$, and ${c}_{2}$) are significant, then social capital exerts a mediating effect in the impact of multiple shocks on households coping strategies. If ${a}_{1}$ is significant and at least one of ${b}_{1}$ or ${c}_{2}$ is not significant, then a bootstrap test is required to further test whether the indirect effect (${b}_{1}{\times c}_{2}$) is significant. If the indirect effect is significant, this indicates a mediating effect of social capital on the effect of multiple shocks on households’ coping strategies. Based on the existence of the mediating effect, we continue to test the significance of ${c}_{1}$. If ${c}_{1}$ is not significant, this indicates a full mediating effect, and if ${c}_{1}$ is significant, this indicates a partial mediating effect.

## Results

### Regression results of multiple shocks, social capital, and households’ coping strategies

The baseline regression results are presented in Table 5. The Hausman test rejects the original hypothesis at a 5% significance level, indicating that the fixed effects model outperforms the random effects mode. Since annual dummy variables are not significant, individual fixed effects models are employed to estimate Eqs. (13)–(15), presented in models 1–3. Models 4–6, using the mixed effects model, are provided for reference.

**Table 5:** Results of baseline regression estimation (n = 2740).

Variable	Model 1	Model 2	Model 3	Model 4	Model 5	Model 6
	Coping strategy	Social capital	Coping strategy	Social capital	Coping strategy	Social capital
Number of multiple	0.411***	$-$0.054*	0.416***	0.477***	− 0.001	0.478***
shocks	(0.033)	(0.003)	(0.033)	(0.017)	(0.002)	(0.017)
Social capital	–	–	0.744*	–	–	0.412*
	–	–	(0.402)	–	–	(0.211)
Age	–	− 0.001	–	–	− 0.002***	–
	–	(0.001)	–	–	(0.002)	–
Education level	0.004	0.004*	− 0.003	− 0.002	0.005***	− 0.005
	(0.024)	(0.002)	(0.024)	(0.009)	(0.001)	(0.009)
Number of household labor force	–	0.010**	–	–	0.013***	–
	–	(0.005)	–	–	(0.002)	–
Household arable land status	0.006	0.002	0.005	− 0.002	0.003***	− 0.001
	(0.025)	(0.002)	(0.024)	(0.012)	(0.001)	(0.012)
Household assets ownership	1.166	0.143**	1.309*	0.385	0.213***	0.518
	(0.723)	(0.063)	(0.726)	(0.346)	(0.034)	(0.352)
Formal insurance	–	0.007	–	–	0.024***	–
	–	(0.011)	–	–	(0.006)	–
Household debt	− 0.277**	–	− 0.214	− 0.135**	–	− 0.096
	(0.126)	–	(0.130)	(0.061)	–	(0.064)
Bank loan	–	− 0.008	–	–	0.018	–
	–	(0.019)	–	–	(0.011)	–
Constants	0.376	0.248***	0.262	0.218**	0.211***	0.178*
	(0.230)	(0.053)	(0.238)	(0.096)	(0.020)	(0.098)
Within R^2^	0.157	0.029	0.160	–	–	–
R^2^	–	–	–	0.228	0.117	0.229

(1) The estimation results of the random effects model are not shown due to space limitations. (2) The LM test rejected the hypothesis of no individual random effects at a 1% significance level, indicating that between random and mixed effects models, the random effects model should be chosen. Combined with the results of the Hausman test, we infer that the fixed effects model is more appropriate than the mixed effects model. (3) Standard errors are in parentheses. (4) ^***^ indicates significance at the 1% level, ^**^ indicates significance at the 5% level, ^*^ indicates significance at the 10% level. (5) Models 1, 2, and 3 are individual fixed effects models, and models 4, 5, and 6 are mixed effects models. (6) The dependent variable of models 1, 3, 4, and 6 is coping strategies, and the dependent variable of models 2 and 5 is the social capital score.

Model 1 demonstrates a significant increase in households’ coping strategies due to the incidence of multiple shocks, validating H1. Model 3 reaffirms this effect even after controlling for social capital, supporting H1. Moreover, social capital significantly increase coping strategies, confirming H2. A 1% increase in the frequency of social capital increased the likelihood of households’ coping strategies by 0.74% (${c}_{2}=0.744$).

Combining models 1–3, it can be inferred that social capital exerts a mediating effect on multiple shocks in terms of households’ coping strategies (H3). Social capital accounts for 9.8% of the total effect. Additionally, household asset ownership significantly increases coping strategies, suggesting that richer asset ownership correlates with a higher likelihood of adopting flexible coping strategies.

In addition, we notice that multiple shocks directly affect social capital. A 1% increase in the frequency of the multiple shocks decreased the likelihood of social capital by 0.05% (${b}_{1}=-0.054$).

### Robustness tests

From a methodological perspective, multiple shocks like natural disasters can serve as natural experiments that allow researchers to effectively define treatment and comparison groups to identify causal relationships. This aligns with exploiting exogenous natural events (i.e., rainfall amounts) as instruments to identify human responses to income shocks, as is commonly pursued in the development economics literature. We conduct the following four robustness tests.

*Measure estimation bias* The effects of multiple shocks and social capital on household coping strategies were estimated using an individual fixed effects model above. Using this model solves the problem of omitted variables that do not vary over time but vary across individuals, but there may still be the problem of omitted variables that vary over time, resulting in estimation bias. Therefore, drawing on the Altonji^56^ and Bellows^57^ methodology, a robustness test was conducted using observable variables to calculate the likelihood of unobservable variables causing biased estimates. Firstly, two regressions are conducted, one without adding control variables over or with only a few control variables and the other with all control variables. The key explanatory coefficients ${\widehat{\beta }}^{R}$ and ${\widehat{\beta }}^{F}$ in the two sets of regressions are computed respectively ($R$ stands for the group of a restricted set of control variables, and $F$ stands for the group of a full set of control variables). Next, the $Ratio$ value is calculated by bringing ${\widehat{\beta }}^{R}$ and ${\widehat{\beta }}^{F}$ into the equation: $Ratio = \left| {\hat{\beta }^{F} /(\hat{\beta }^{R} - \hat{\beta }^{F} )} \right|$. The above formula shows that the smaller the difference between ${\widehat{\beta }}^{R}$ and ${\widehat{\beta }}^{F}$, the larger the $Ratio$ value, and the smaller the effect of the observable variables on the dependent variable, i.e., the larger the estimation bias caused by the unobservable variables. The larger $|{\widehat{\beta }}^{F}|$ is, the larger the $Ratio$ value is, and the larger the effect of the unobservable variables on the dependent variable. However, it is important to note that the larger the $Ratio$ value, the less likely the biased estimates are due to omitted variables. This is because to change the current estimates, more omitted variables should be included in the existing model, and the explanatory power of these omitted variables on the dependent variable would need to become greater. This possibility decreases as the value of the $Ratio$ increases.

Two regressions with a restricted set of control variables and two regressions with a full set of control variables are constructed for the dependent variable of household coping strategies^58^. The former consists of regressions one and two: Regression one introduces the key independent variable (multiple shocks) without the control variables; Regression two introduces multiple shocks and total assets and years of education. The latter contains regression three and regression four: Regression three contains all the control variables from equation three; Regression four contains all the control variables from equation three and the province dummy variables. Then, this paper utilizes the mixed regression model to sequentially estimate the coefficients of the key independent variable (multiple shocks) in the above four regressions and calculate the $Ratio$ values separately. Based on the above principle, this paper calculates and displays the $Ratio$ values in Table 6.

**Table 6:** Measurement of estimation bias.

	Restricted set of control variables	Full set of control variables	Ratio value
Condition 1	Regression one	Regression three	353.377
Condition 2	Regression two	Regression three	332.040
Condition 3	Regression one	Regression four	41.845
Condition 4	Regression two	Regression four	33.450

Due to a larger value of $Ratio$, the possibility of bias in estimation due to omitted variables is very low in mixed regressions. The benchmark regression with fixed effects model also solves the problem of omitted variables that do not vary from time to time, which is better than using the mixed regression model, so it can be considered that omitted variables are even less likely to cause estimation bias in the benchmark regression with fixed effects model. Therefore, the results of the benchmark regression are credible.

The results show that the $Ratio$ values calculated under the four conditions range from 33.450 ~ 353.377, with a mean value of 190.178. In other words, to improve the existing estimation results, the effect of the omitted variables would have to be at least 33.450 times as large as the effect of the existing control variables, which is highly unlikely. This result proves the robustness of this paper’s baseline regression estimation results.

*Adjust social capital score* To address endogeneity arising from social capital and coping strategies, the indicators of informal borrowing and loans and gift income are excluded, redefining the original six dimensions into four dimensions, and redistributing the weights among the remaining dimensions^59^. The same individual fixed effects model is used to estimate Eqs. (13)–(15) separately, and the estimated results (Table 7) are consistent with the baseline regression, confirming that the conclusions are robust.

**Table 7:** Robustness test for replacing key independent variables.

Variable	Model 7	Model 8	Model 9
	Coping strategy	Social capital	Coping strategy
Number of multiple	0.411***	− 0.012***	0.419***
shocks	(0.033)	(0.003)	(0.034)
Social capital score	–	–	0.624*
	–	–	(0.339)
Control variable	YES	YES	YES
Within R^2^	0.157	–	0.157
Between R^2^	–	0.091	–
Model	FE	RE	FE

(1) Standard errors are in parentheses. (2) ^***^ indicates significance at the 1% level, ^**^ indicates significance at the 5% level, ^*^ indicates significance at the 10% level. (3) RE means random effects model; FE means fixed effects model.

*Balancing tests* Considering that the data used in the baseline regression are unbalanced panel data, the sample in two waves is inconsistent and may cause bias in the estimation results. We extract a balanced panel subsample (913 observations each year) from the unbalanced panel sample, and apply the individual fixed effects to estimate Eqs. (13)–(15). The estimation results (Table 8) are consistent with the baseline regression, indicating that the conclusions are robust.

**Table 8:** Robustness test for balancing panel data (n = 1826).

Variable	Model 10	Model 11	Model 12
	Coping strategy	Social capital	Coping strategy
Number of multiple	0.411***	− 0.005*	0.416***
shocks	(0.033)	(0.003)	(0.033)
Social capital score	–	–	0.744*
	–	–	(0.402)
Control variable	Yes	Yes	Yes
Observations	1 826	1 826	1 826
Within R^2^	0.157	0.029	0.160

(1) Values in parentheses are standard error. (2) ^***^ means significant at the 1% level, ^**^ means significant at the 5% level, ^*^ means significant at the 10% level.

*Adjustment the model* The dependent variable of this paper, households’ coping strategies, is a non-negative discrete random variable. Some households do not have any coping strategies, and the number of coping strategies is zero, while others’ coping strategy is equal to or greater than 1, which does not conform to the normal distribution required by classical linear regression. Therefore, this paper considers a Poisson Regression count data model to re-estimate the parameters^60,61^. The estimation results are consistent with the baseline regression (Table 9), confirming that the conclusions are robust.

**Table 9:** The Poisson Regression results.

Variable	Model 13	Model 14	Model 15
	Coping strategy	Social capital	Coping strategy
Number of multiple	0.289***	− 0.009*	0.289***
shocks	(0.014)	(0.006)	(0.007)
Social capital score	–	–	0.358**
	–	–	(0.140)
Control variable	Yes	Yes	Yes
Prob > chi2	456.11	544.864	1412.478
Pseudo R2	0.155	0.015	0.156
Observations	2740	2740	2740

(1) Standard errors are in parentheses. (2) ^***^indicates significance at the 1% level, ^**^indicates significance at the 5% level, ^*^ indicates significance at the 10% level.

### Heterogeneity analysis of different shocks and households

We address two key inquiries. Firstly, while household coping strategies may effectively handle small to medium-sized idiosyncratic shocks, concerns arise regarding households’ resilience against large, compounded multiple shocks occurring simultaneously^62^. The increasing frequency and intensity of shocks can overwhelm existing coping strategies, surpassing resilience. The mediating role of social capital varies based on the shock type. Secondly, social capital is unevenly distributed among rural households, with disparities in access and benefits^20,63^. Notably, social capital may not be equally accessible to disaster victims, particularly those with low incomes. Although social capital increases households’ coping strategies, multiple shocks decrease households’ social capital directly (in Table 5 model 2). When households experience multiple shocks, the available social capital they can mobilize could be scarce, leaving households’ livelihood in a vulnerable state. Understanding household responses to shocks and the dynamics of social capital’s opportunities and limitations informs strategies for enhancing household resilience.

*Impact of different shocks on households’ social capital* Referring to the literature^64,65^, we divide shocks into three categories: natural disaster shocks, social shocks and economic shocks. Natural disasters, as homogenous risks with a broader scope of impact, significantly negatively affect social capital at the 1% level (Table 10). However, social shocks and economic shocks show no significant effect on social capital.

**Table 10:** Estimation results for different types of shocks.

Variable	Model 16	Model 17	Model 18
	Social capital	Social capital	Social capital
Number of natural disaster shocks	− 0.010***	–	–
	(0.004)	–	–
Number of social shocks	–	0.002	–
	–	(0.008)	–
Number of economic shocks	–	–	− 0.001
	–	–	(0.009)
Sample size	2740	2740	2740
Within R^2^	0.032	0.025	0.025

(1) Standard errors are in parentheses. (2) ^***^indicates significance at the 1% level, ^**^indicates significance at the 5% level, ^*^ indicates significance at the 10% level.

*Heterogeneity of different shocks on different coping strategies* It is also important to study households’ shock coping strategies to gain a deeper understanding of the impact of shocks on household resilience. As mentioned before, different shocks may impact households’ coping strategies differently. Therefore, this paper further categorizes shocks into natural disaster shocks and unnatural disaster shocks, categorizes coping strategies into self-insurance and risk-sharing strategies, and discusses the impacts of the different shocks on households’ different coping strategies^34,35^. In this study, reducing consumption, using savings, working outside the home, and selling assets are self-insurance strategies, and borrowing money is risk-sharing strategy. The results in Table 11 show that self-insurance strategy increases significantly in both natural and unnatural disasters. Unnatural disasters significantly increase risk-sharing strategies, but natural disaster shocks do not significantly increase risk-sharing strategies.

**Table 11:** Estimation results for different types of shocks on different coping strategies.

Variable	Model 19	Model 20	Model 21	Model 22
	Self-insurance strategies	Risk-sharing strategies	Self-insurance strategies	Risk-sharing strategies
Number of natural disaster shocks	0.029***	0.02	–	–
	(0.010)	(0.012)	–	–
Number of unnatural disaster shocks	–	–	0.098***	0.042**
	–	–	(0.014)	(0.016)
Sample size	2740	2740	2740	
Within R^2^	0.013	0.004	0.055	0.008

(1) Standard errors are in parentheses. (2) ^***^indicates significance at the 1% level, ^**^indicates significance at the 5% level, ^*^indicates significance at the 10% level.

*Heterogeneity of households with different livelihoods* Households are classified into four types based on the proportion of non-farm income to total income^62^, including households fully engaged in agriculture (non-farm income < 10%), primarily engaged in agriculture (10% ≤ non-farm income < 50%), less engaged in agriculture (50% ≤ non-farm income < 90%), and almost not engaged in agriculture (non-farm income is ≥ 90%). Table 12 indicates that when multiple shocks occur, a higher proportion of non-farm income facilitates more flexible coping strategies, especially for households almost not engaged in agriculture, where social capital has a fully mediating effect.

**Table 12:** Estimation results for different livelihood types of farm households (n = 2740).

Variable	Fully engaged in agriculture			Mostly engaged in agriculture			Less engaged in agriculture			Fully not engaged in agriculture		
	Model 23	Model 24	Model 25	Model 26	Model 27	Model 28	Model 29	Model 30	Model 31	Model 32	Model 33	Model 34
	Coping strategy	Social capital	Coping strategy	Coping strategy	Social capital	Coping strategy	Coping strategy	Social capital	Coping strategy	Coping strategy	Social capital	Coping strategy
Number of multiple shocks	0.469***	0.004	0.464***	0.104	0.001	0.104	0.454***	0.001	0.454***	0.480***	− 0.019**	0.485***
	(0.044)	(0.003)	(0.044)	(0.116)	(0.006)	(0.116)	(0.034)	(0.003)	(0.033)	(0.028)	(0.008)	(0.028)
Social capital score	–	–	0.485	–	–	− 0.479	–	–	0.483	–	–	0.720**
	–	–	(0.637)	–	–	(2.128)	–	–	(0.387)	–	–	(0.301)
Control variable	Yes	Yes	Yes	Yes	Yes	Yes	Yes	Yes	Yes	Yes	Yes	Yes
Sample size	313	313	313	693	693	693	827	827	827	907	907	907
Within R^2^	–	–	–	0.013	0.066	0.013	–	–	–	–	0.123	–
Between R^2^	0.278	0.165	0.276	–	–	–	0.211	0.111	0.212	0.289	–	0.293
Model	RE	RE	RE	FE	FE	FE	RE	RE	RE	RE	RE	RE

(1) Standard errors are in parentheses. (2) ^***^indicates significance at the 1% level, ^**^indicates significance at the 5% level, ^*^ indicates significance at the 10% level. (3) The results of control variable estimation are omitted for statistical convenience. (3) RE means random effects model; FE means fixed effects model.

## Discussion

This study delves into multiple shocks, social capital, and households’ coping strategies in underdeveloped rural areas of China. Firstly, prevalent shocks such as drought, illness, and the high agricultural input costs prompt households to primarily reduce consumption. The study identifies idiosyncratic and covariate shocks, emphasizing their severe impact in low-income countries with limited credit markets and social insurance mechanisms. These shocks force households to cut essential expenditures, such as nutritious food or education^25^, contributing to short-term hardships and potentially long-term welfare undermining.

Secondly, the results demonstrate that multiple shocks and social capital significantly positively impact households’ coping strategies, supporting theoretical hypotheses. The study’s significant contribution lies in revealing the mediating role of social capital, explaining 9.8% of the impact of multiple shocks on coping strategies. This aligns with previous research^20,66,67^, attributing this effect to strengthened traditional functions in China, characterized by strong social ties and family-based connections. Modernization, technological advancements, and expanded social networks further enrich coping strategies.

Another possible explanation is that the form of social capital has changed at present. With China’s modernization of agriculture and rural areas, the enrolment of farmers’ children in universities, and the movement of capital to the countryside, households are no longer confined to their original social networks but undergo a vertical extension of social networks. Some households have upper social capital, such as government agencies and financial institutions, which are conducive to promoting well-being, such as economic opportunities, employment opportunities, and human capital^68,69^. These resources will enrich households’ coping strategies when they face multiple shocks. In addition, with the development of information technology, computers and smartphones have been popularized by households in undeveloped rural areas, and the scope of interactions of households has been expanding^70^. This has greatly broadened households’ access to knowledge, enriching farmers’ risk management. As China’s urban–rural integration develops, the traditional functions of social capital have been strengthened and given rise to new forms. A recent study in China supports this conclusion^71^.

Thirdly, heterogeneity analysis uncovers the differential effects of shocks on social capital. Natural disasters significantly diminish the informal function of social capital. Natural disaster shocks do not significantly increase risk-sharing strategies based on social capital. which is consistent with Dercon^72^ and Gao et al.^73^. A possible reason is that natural disasters are large-scale shocks that imply a wide range of damage to different farm households. In the case of widespread farmers’ exposure to natural disaster shocks, social capital is severely impaired, and informal insurance limits (our theoretical model in part 2 also demonstrates that). Coping strategies that rely on social capital have limitations in the face of covariate natural disasters. Kinship networks and informal, community-based resources may be strained if several members require support simultaneously^74^. Research suggests that some informal moneylenders may be unable to provide sufficiently large loans to numerous afflicted households^75^. In contrast, the damage experienced by individual households can have a significant idiosyncratic component that is insurable through informal risk-sharing arrangements. For example, When a household’s labor efficiency is reduced due to health shocks, family and friends can help with production during the farming season and can also give financial support for the education of the shocked family^76^. However, a natural disaster can exert a region-wide covariate shock (i.e., all households in the region suffer from flood), and social capital tends to be considered ineffective against such covariate shocks.

Another heterogeneity in the population suggests that social capital exerts a full mediating effect in households that are not engaged in agriculture but not in others. A person’s position in exchange networks is a function of one’s ability to regularly reciprocate, which, in turn, is a function of one’s position in the community wealth ladder. Interpersonal or interhousehold differences, especially in human, financial, and political capitals, may easily generate unequal access to social capital and other livelihood resources. Being resource-poor may lead to exclusion from exchange networks and from accessing social capital resources, which is considered one of the limitations of social capital. According to Lin’s theory^77^, low-income farmers lack high-quality social capital, and the poor can mobilize fewer social resources than higher-income farmers. Hence, the returns to social capital for the poor are also likely to be lower than those to the rich. An ethnographic study based on the poorest people in Tanzania, Africa, found that social relations, collective action, and regional organizations also continually and structurally exclude the poor^78^. In multiple shocks cases, social capital is unfavorable to lower-income households engaged in agriculture, resulting in relative deprivation between groups, and social capital is not the capital of the poor.

## Conclusions and policy recommendations

Farm households worldwide face increasing shocks, necessitating resilience for sustainable livelihoods. Studying the role of social capital amidst multiple shocks is particularly meaningful because these shocks impose severe stress on governments and communities, testing not only government effectiveness but also the robustness of social institutions. While the importance of social capital for resilience is now acknowledged, studies often lack detailed characterization and contextualization and may fail to adequately assess the sustainability of the process. Furthermore, studies on resilience, shock management policy, and practices have yet to fully embrace informal support against livelihood shocks. This study utilizes a China-based case to deepen understanding of the relationships between multiple shocks, social capital, and coping strategies. Conclusions include the prevalence of specific shocks, the mediating effect of social capital, and the differential impact on social capital based on shock types and household engagement in agriculture.

The policy implications based on our research findings are threefold. First, we argue that the inherent potentials embedded within social capital will only be tapped and become a tool to promote positive development outcomes if policymakers and development practitioners acknowledge their role, mainstream them in the country’s development strategy, and complement them with genuine interventions for more inclusive development. Second, our results show the need for more support and interventions to assist rural households in preventing and mitigating the effects of natural disasters. Backing and complementing informal arrangements through external interventions in areas where they cannot be insured can be vital. As agricultural production activities are highly dependent on weather conditions, public investment in the expansion and rehabilitation of irrigation and drainage infrastructure would be necessary to reduce the dependence of agricultural production activities on weather conditions and the cost of agricultural inputs. Third, it is essential to note that a balanced combination of social capital and government support makes a difference in tackling the vulnerability of low-income people. Individuals and households on the wealth ladder can be exposed differently to the same type of shocks and face different constraints and opportunities in mobilizing resources to cope with them. This strongly signals social development policymakers and practitioners to consider resource-poor households in their intervention.

Modern China represents a special case of rural development that is unique worldwide due to the scale and speed of changes affecting its huge and populous rural areas. The China-based case is beneficial in better understanding the complexity and specificities of social capital in vulnerable rural areas. Although the above conclusions are drawn in the Chinese context, they have important implications for other developing countries, where farmers are situated in a social network of relationships and are challenged by multiple shocks. While this study unveils important insights, limitations include sample loss and the inability to track structural changes in social capital. Future research can explore social capital transformation in anti-poverty efforts and investigate policy tools to create and expand household social capital.

## Data Availability

The datasets used and/or analysed during the current study available from the corresponding author on reasonable request. Requests to access the datasets should be directed to huangjiaqi@caas.cn.
